# Snail-Family Proteins: Role in Carcinogenesis and Prospects for Antitumor Therapy

**DOI:** 10.32607/actanaturae.11062

**Published:** 2021

**Authors:** M. A. Yastrebova, A. I. Khamidullina, V. V. Tatarskiy, A. M. Scherbakov

**Affiliations:** Institute of Gene Biology, Russian Academy of Sciences, Moscow, 119334 Russia; Blokhin National Medical Research Center of Oncology, Moscow, 115478 Russia

**Keywords:** transcription factors, Snail, Slug, epithelial-mesenchymal transition, bbb, mesenchymal-epithelial transition, breast cancer

## Abstract

The review analyzes Snail family proteins, which are transcription factors involved in the regulation of the epithelial-mesenchymal transition (EMT) of tumor cells. We describe the structure of these proteins, their post-translational modification, and the mechanisms of Snail-dependent regulation of genes. The role of Snail proteins in carcinogenesis, invasion, and metastasis is analyzed. Furthermore, we focus on EMT signaling mechanisms involving Snail proteins. Next, we dissect Snail signaling in hypoxia, a condition that complicates anticancer treatment. Finally, we offer classes of chemical compounds capable of down-regulating the transcriptional activity of Snails. Given the important role of Snail proteins in cancer biology and the potential for pharmacological inhibition, Snail family proteins may be considered promising as therapeutic targets.

## INTRODUCTION

During metastasis, tumor cells acquire a locomotor phenotype, enter the bloodstream, and form premetastatic niches in target organs. The colonization of metastatic niches by tumor cells leads to the formation of secondary tumors [1, 2]. The process by which highly differentiated polarized epithelial cells acquire a locomotor phenotype of mesenchymal cells is called the epithelial-mesenchymal transition (EMT) [3]. The key role in the regulation of this process is played by Snail family proteins, which are transcription factors that control the expression of the genes whose products determine the EMT phenotype(s) and, ultimately, the progression of neoplasms [4]. Over the past 15 years, new-generation antineoplastic agents have been developed. Antitumor therapy has become targeted and has focused on the individual mechanisms that regulate the vital activity of tumor cells. Clinical practice has been expanded by the introduction of protein kinase inhibitors, modulators of the death/survival balance, proteasome inhibitors, etc., which yield significant therapeutic results in certain groups of patients [5, 6, 7, 8]. Along with classic chemotherapy regimens, personalized approaches based on the biological characteristics of a particular neoplasm have been tested. These approaches are especially important in the development of optimal treatment regimens for patients with metastasis.

Despite the progress achieved in understanding the mechanisms of metastasis, there are still no effective antimetastatic drugs; therefore, the investigation of molecules that reduce the metastatic potential of a tumor remains topical.

The review discusses the signaling pathways of Snail family proteins, their role in maintaining an aggressive behavior of a tumor cell, and prospects for the pharmacological regulation of EMT in clinical practice.

## METASTASIS AND EMT

Since the first description of the EMT phenomenon [3], more light has been shed on its key mechanisms. The main EMT criteria include changes in the expression of the marker genes of epithelial and mesenchymal cells, as well as the changes taking place in the morphology of cells and the increase in their migration ability. Cytokines, growth factors, and extracellular matrix (ECM) molecules activate the signaling pathways that trigger the EMT program. These pathways are mediated by a number of transcription factors (Slug, Snail, ZEB1/2, Twist1/2, etc.) that bind to the regulatory regions of target genes. Regulation of EMT by the products of these genes leads to the inhibition of epithelial markers (E-cadherin, claudins, occludin, etc.) and activation of mesenchymal markers (vimentin, fibronectin, N-cadherin, etc.). Mesenchymal cells exhibit enhanced motility, invasiveness, resistance to apoptosis, and production of ECM components [9, 10].

After acquiring a mesenchymal phenotype, tumor cells are able to migrate from the epithelial layer, via the bloodstream, and, after reaching a metastatic niche, return to their initial phenotype through the mesenchymal-epithelial transition (MET), which leads to the formation of metastases. There are studies that have explored the mechanisms of MET regulation, including the dynamic regulation of the factors that induce MET during the metastatic cascade. A gradual decrease in Snail expression in tumor cells during colonization, which is due to inhibition by microRNAs, causes MET induction: in particular, miR-34 and miR-200 inhibit Snail and ZEB1/2 transcription factors [11, 12, 13]. However, it is not entirely clear whether MET is an actively regulated process triggered by certain signaling molecules, or whether it occurs passively in the absence of factors that stimulate and maintain EMT in the metastatic site, as compared to the primary tumor.

EMT occurs in many processes in embryonic (mesoderm formation, migration of neural crest cells, left-right asymmetry determination, and parietal endoderm formation) and postnatal development [14, 15]. In disease, EMT is associated with malignant transformation, tumor progression, and fibrosis development. There are studies of Snail and Slug proteins as EMT regulators during tumor progression where they are involved in the regulation of cell survival and proliferation, invasion, and metastasis [16, 17, 18], as well as regulate energy metabolism and maintain resistance to therapy [19].

The new EMT classification includes four stages: epithelial, early hybrid, late hybrid, and mesenchymal. Snail activity was shown to increase starting from the early hybrid stage, while changes in the shape of cells, from round to elongated, occur only at the late hybrid stage. These changes are accompanied by a gradual loss of intercellular adhesion [20].

## STRUCTURE OF Snail FAMILY PROTEINS

**Fig. 1:**
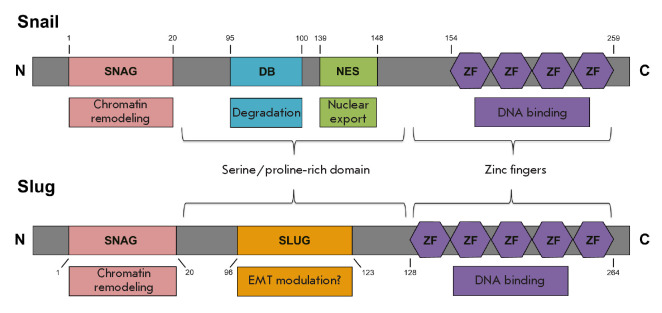
The structure of Snail and Slug proteins

Snail family proteins, Snail/*SNAI1 *and Slug/*SNAI2*, are transcriptional repressors [21]. These proteins contain a highly conserved C-terminal region that includes four (Snail) and five (Slug) zinc fingers and is involved in the binding of the proteins to the target gene promoters containing the E-box sequence. The N-terminal regions contain the evolutionarily conserved SNAG domain required for transcriptional repression and capable of binding methyltransferases and histone deacetylases [4]. Despite the similarity of the N- and C-terminal regions of Snail and Slug, the central proline-rich regions, which mediate ubiquitination and the proteolytic degradation of these proteins, are different. Snail contains a protein destruction box (DB) domain and a nuclear export signal (NES) domain, while Slug comprises a specific SLUG domain. The SNAG and SLUG domains of the Slug protein are required for the repression of the E-cadherin gene promoter. The SLUG domain interacts with the CtBP1 corepressor, while the SNAG domain interacts with the NCoR corepressor [22]. Interestingly, the SNAG domain is required for EMT induction, while the SLUG domain probably negatively regulates the Slug-mediated EMT [23] (*Fig. 1*).

The functional activity of the proteins is determined by their structure, configuration, and post-translational modifications [24].

## POST-TRANSLATION MODIFICATIONS OF Snail FAMILY PROTEINS

Snail is a labile protein whose half-life is less than 4 h [25]. Like many proteins, Snail undergoes various post-translational modifications that affect its stability, intracellular localization, and transcriptional activity. There are two Snail phosphorylation sites: one controls the proteolysis of the protein in the proteasome, and the other determines its intracellular localization. Glycogen synthase kinase-3β (GSK-3β) binds to Snail and phosphorylates it, causing export of the protein from the nucleus to the cytoplasm. Subsequent GSK-3β-mediated phosphorylation in the cytoplasm promotes the binding of Snail to E3-ubiquitin ligase β-TrCP and degradation of Snail in the proteasome [26]. Both phosphorylated and non-phosphorylated Snail forms can bind to ubiquitin ligase FBXL14, which also leads to proteasomal degradation of Snail. DUB3 deubiquitinase was shown to be able to prevent the degradation of Snail in the proteasome, thereby stabilizing it [27]. Stabilization of Snail in the nucleus also involves protein kinase PAK1 that enables Snail phosphorylation at the serine residue in position 246. In turn, Snail phosphorylation by protein kinase A (PKA) at serines 11 and 92 enhances Snail transactivation [28].

Stability of the Slug transcription factor is similarly regulated and depends on phosphorylation by protein kinase GSK-3β. The Slug phosphorylation sites (Ser-4 and 88) have been identified. Phosphorylation of serine 4 is required for a Slug-mediated induction of EMT [23].

Stabilization of Snail/Slug involves, apart from phosphorylation by protein kinases, histone acetyltransferases (HATs) that provide nuclear localization of Snail/Slug and their interaction with co-activators [29]. E3 ubiquitin ligase A20 monoubiquitinates Snail at three lysine residues, which reduces the affinity of Snail for GSK-3β and maintains its nuclear localization, facilitating breast cancer (BC) cell EMT induced by transforming growth factor β (TGF-β1). A20 knockdown or increased Snail expression with replacement of monoubiquitinated lysine residues by arginine prevents metastasis in BC models [30].

## TARGETS OF Snail FAMILY TRANSCRIPTION FACTORS

Slug and Snail proteins, despite the significant (~70%) homology of their amino acid sequences, are functionally different. For example, Snail activity is necessary in early embryogenesis, because mouse embryos with knockout *snai1 *die at the gastrulation stage, due to impaired formation of the mesoderm layer, where cells retain epithelial features such as polarity, tight intercellular junctions, and E-cadherin expression [31].* Snai2 *knockout mice are viable, but they have defects in neural crest cell formation and mesoderm formation [32]. Both Snail and Slug are required for osteogenesis, chondrogenesis [33], and somitogenesis [34].

Snail and Slug are necessary for the regeneration of adult tissues; in particular, for wound healing [15]. The key role in this process is played by Slug that is controlled by the epidermal growth factor (EGF) secreted during healing [35]. In *snai2 *knockout mice, there is no migration of keratinocytes into the wound while K6 and Ki-67 proliferation markers and high E-cadherin and K8 levels are retained [36].

In a human colorectal cancer model, ChIP-seq experiments demonstrated that the Snail transcription factor mainly binds to regions located upstream of the transcription start site (within 1 kbp), as well as in intergenic regions and introns distal to the promoter. Therefore, Snail controls transcription mainly through binding to distant regulatory DNA elements [37]. Snail was found to predominantly bind to the genes responsible for differentiation, morphogenesis, organogenesis, signal transduction, and cell junctions, which is in good agreement with its known biological functions [37]. In triple negative BC cells, two more Snail binding sites were identified: the TAL/GATA1 and TGG RREB1/ RUNX2/PAX4 motifs, which provide more specific recognition of target genes compared to other transcription factors [38].

Snail and Slug can act both as transcriptional repressors and as activators of transcription of genes encoding mesenchymal proteins: N-cadherin, vimentin, fibronectin, etc. [39, 40]. Snail can also induce transcription by interacting with the transcription factors EGR1 and SP1 [41].

**Fig. 2:**
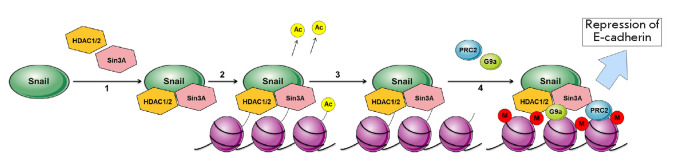
Snail-mediated repression of E-cadherin. 1 – Formation of the repressor complex (Snail, HDAC1/2, Sin3A); 2 – deacetylation of H3 and H4 histones; 3 – binding of the PRC2 inhibitory complex and methyltransferase G9a; 4 – DNA hypermethylation. Adapted from [42]

The Snail-mediated mechanism of gene expression repression was studied in detail in the case of E-cadherin, an epithelial cell marker (*Fig. 2*).

The SNAG domain of the Snail protein interacts with the Sin3A protein and the histone deacetylases (HDAC) 1 and 2. The resulting complex binds to the E-box region in the E-cadherin gene (*CDH1*) promoter, which leads to de-acetylation of histones H3 and H4. This modification facilitates the binding of the inhibitory complex PRC2 and histone methyltransferase G9a: the second act of E-cadherin expression inhibition occurs via DNA hypermethylation. After the initial suppression of E-cadherin, Snail induces expression of the transcription factor ZEB1, which further inhibits E-cadherin expression, but through a PRC2-independent mechanism, the details of which are still unknown [42].

Snail/Slug-dependent transcription leads not only to the repression of E-cadherin but also to the disassembly of desmosomes and tight intercellular junctions due to repression of occludin, claudin 3, 4, and 7, and desmoplakin genes [43, 44]. Snail and Slug also increase synthesis of matrix metalloproteinases (MMPs), thereby promoting degradation of ECM components [45, 46].

Changes in cell motility during EMT and the development of the locomotor phenotype are associated with the activity of Rho family proteins; small GTPases Rac1, RhoA, RhoV, and Cdc42, which control actin dynamics [47]. Rac1 regulates the TGF-β-dependent activation of Snail: knockdown of Rac1 decreases the activity of Snail and MMP9 [48]. In contrast, inhibition of RhoA increases the Snail level [49]. RhoV, together with Snail, induces Slug in EMT during embryonic development [50]. The increase in the motility of pancreatic cancer cells associated with an elevated Snail level depends on Rac1 [45], and an increase in the Slug level leads to the suppression of ROCK1/2 [46]. Suppression of Snail significantly reduces cell motility because of the lower activity of Cdc42 and increased activity of RhoA [51]. Thus, both proteins, Snail and Slug, are controlled by the small GTPases responsible for cell motility and can regulate GTPase activity, enabling a coordination of changes in cell phenotypes during embryogenesis and tumor progression.

Snail plays an important role in the cell cycle and in cell survival. During embryonic development, Snail represses the transcription of the cyclin D2 gene and increases the expression of the p21Cip1/WAF1 gene in order to regulate early-to-late G1 phase transition. An increase in the expression of cyclin-dependent kinases CDK4/6 promotes Snail stabilization through DUB3- mediated deubiquitination [27]. In renal epithelial cells (MDCK line) stably expressing exogenous Snail, about 90% of the cells remain in the G0/G1 phase after 72-h incubation. Overexpression of Snail decreases CDK4, and phosphorylation of Rb and increases the p21^Cip1/WAF1^ level [52]. Thus, Snail can be used to delay or stop the transition of cells in the cell cycle.

Slug is also involved in the regulation of cell-cycle phase alteration. Slug was shown to act in functional cooperation with cyclin D1. Slug knockdown in the MDA-MB-231 triple negative BC cell line reduces the rate of cell proliferation, probably due to a decrease in the cyclin D1 level [53]. According to another study, induced Slug expression can lead to the inhibition of cyclin D1 and arrest of prostate cancer cells in the G0/G1 phase. Thus, the role of Slug varies in cells of different tissue origins [54].

Snail regulates cell survival through decreasing the serum concentration in the culture medium by activating the MAPK (Mek/Erk) and PI3K signaling pathways. Snail and Slug suppress the expression of several pro-apoptotic factors at the transcriptional level; in particular p53, BID, caspase 6, PUMA/BBC3, ATM, DFF40 (DNA fragmentation factor), and PTEN (phosphatase in the PI3K cascade) [52, 55, 56, 57]. Interestingly, the Snail protein can directly interact with the tumor suppressor p53, blocking its DNA-binding domain [58].

It is noteworthy that the transcriptional targets of Snail and Slug are similar, but information on mutual regulation of these proteins is insufficient. According to our data, expression of Snail and Slug is interdependent. For example, Snail overexpression in the MDA-MB-231 cell line is accompanied by a sharp decrease in the Slug protein level while Snail inhibition by small interfering RNAs is associated with an increase in the Slug level. Probably, Snail and Slug compensate each other under certain conditions [59].

Various exogenous stimuli can activate Snail-family transcription factors. Below, we provide the results of an analysis of the main signaling pathways that regulate Snail and Slug.

## REGULATION OF Snail-FAMILY PROTEINS DURING EMT

**Fig. 3:**
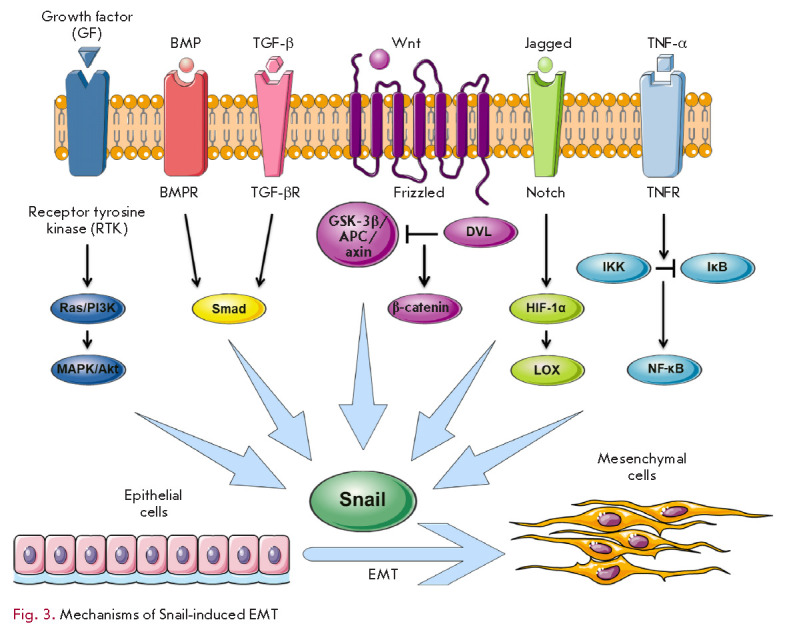
Mechanisms of Snail-induced EMT

EMT is a dynamic process that can be initiated by ECM proteins and secreted, soluble growth factors, such as the epidermal growth factor (EGF), hepatocyte growth factor (HGF), fibroblast growth factor (FGF), bone morphogenetic proteins (BMPs), TGF-β, Wnt, Notch, tumor necrosis factor α (TNF-α), and cytokines [60, 61]. Many of these signaling molecules from the tumor cell microenvironment induce the expression of Snail-family proteins (*Fig. 3*).

Signaling cascades initiated by the activation of receptor tyrosine kinases (RTKs) and growth factors cause an increase in the level of Snail, its stabilization, and translocation into the nucleus. MAPK or PI3K signaling cascades cooperate with TGF-β to regulate EMT [62]. Repression of MAPK in some tumor models is sufficient to reduce the expression of Snail and Slug and inhibit EMT [63, 64, 65].

The multifunctional protein TGF-β regulates proliferation, differentiation, and apoptosis. TGF-β acts as a tumor growth suppressor at the early stages of carcinogenesis and promotes the formation of a malignant phenotype at later stages [66]. Snail plays an important role in regulating the response of cells to TGF-β, ensuring their resistance to TGF-β-mediated apoptosis and tumor progression. At later stages, TGF-β induces EMT in a SMAD-dependent manner via Snail. SMAD proteins interact with the *SNAI1 *gene promoter and induce Snail expression, which leads to the repression of E-cadherin and an invasive phenotype [4]. Upon TGF-β-induced EMT, Snail was shown to form a complex with SMAD3/4. This complex binds to E-box regions and SMAD-binding elements in the promoters of the genes encoding intercellular junction proteins and represses these genes [67].

Activation of the Notch signaling pathway induces Snail/Slug-mediated EMT, which promotes BC cell invasion and metastasis [68]. Notch controls Snail expression through two synergistic mechanisms: direct activation of transcription and indirect action through lysyl oxidase (LOX) that stabilizes Snail. Notch recruits the hypoxia-inducible factor 1α (HIF-1α) to the* LOX *promoter, activating this gene [67]. In addition, Jagged1-activated Notch stimulates the Slug repressor and suppresses E-cadherin, which leads to the so-called hybrid (intermediate) EMT phenotype. This phenotype is characterized by a partial increase in the expression of mesenchymal markers and a decrease in the expression of epithelial markers. In this case, there are no significant morphological changes in cells, and there is no complete loss of intercellular junctions [69].

Expression of the *SNAI1 *gene can also be regulated by the nuclear factor NF-κB/p65. TNF-α-activated NF-κB binds to the *SNAI1 *promoter; activation of the transcription of this gene induces EMT [25]. *SNAI1 *expression can also be enhanced through the Akt signaling pathway: the protein kinase Akt1 phosphorylates IKKα, which leads to proteolytic degradation of the inhibitory subunit IκB, release of NF-κB dimers and their translocation into the nucleus, and transactivation of *SNAI1 *[70]. Simultaneous suppression of Snail and NF-κB was shown to increase the sensitivity of BC cells to antiestrogens [71]. A simultaneous influence on these two transcription factors may be of interest for the development of approaches to anticancer therapy.

Activation of the Wnt signaling pathway is accompanied by the inhibition of β-catenin and Snail phosphorylation by GSK-3β, which leads to the accumulation of β-catenin and Snail in the nucleus. β-Catenin, which acts as a transcription factor in its interaction with TCF/LEF, is required for EMT induction in epithelial cells. The synergistic effect of Snail and β-catenin enables tumor cell survival during invasion and metastasis [72].

The MDM2 protein also plays a role in EMT. Increased expression of MDM2 in MCF7 BC cells leads to an epithelial-to-mesenchymal change in their morphology. On the other hand, knockdown of MDM2 in MDA-MB-231 cells changes the cell morphology from mesenchymal to epithelial (MET). In addition, enhanced expression of MDM2 increases the expression of N-cadherin and vimentin and also decreases the expression of E-cadherin at the mRNA and protein levels. Downregulation of MDM2 expression decreases the expression of N-cadherin and vimentin and increases the expression of E-cadherin. MDM2 increases the level of both mRNA and the Snail protein by activating the TGF-β-SMAD signaling pathway. *SNAI1 *knockdown in cells that had entered MDM2-induced EMT was shown to return such cells to their initial epithelial phenotype. Thus, MDM2, like Snail, may be considered a therapeutic target in metastatic BC [73].

It is important that the key EMT-mediating transcription factors can affect the expression of each other. We demonstrated that knockdown of the *TWIST1* and *ZEB1 *genes by small interfering RNAs decreases the Slug protein level, with no opposite effect being observed [59].

## HYPOXIA AND EMT

One of the EMT regulation factors is hypoxia. Tumor growth leads to a deficiency in oxygen and nutrients in the tumor. This “starvation,” on the one hand, inhibits the proliferation of cells and, on the other hand, induces adaptation processes in them, in particular EMT, which enables the tumor cells to migrate to blood vessels. Adaptation of cells to hypoxia involves hypoxia-inducible proteins, such as the HIF-1 transcription factor, a heterodimer composed of the HIF-1α and HIF-1β subunits [74, 75]. Under normoxia conditions, HIF-1α is hydroxylated by prolyl hydroxylase, which leads to the binding of HIF-1α to the Hippel–indau protein (VHL), a ubiquitination marker. The VHL–IF-1α interaction leads to a degradation of HIF-1α in the proteasome. Under oxygen deficiency, the activity of prolyl hydroxylase decreases and HIF-1α fails to undergo rapid degradation because the lack of hydroxylated proline residues stabilizes HIF-1α [76]. HIF-1α accumulates in the cell and dimerizes with HIF-1β, forming an active transcription factor that is translocated into the nucleus, binds there with the hypoxia-responsive element (HRE) sites on DNA and activates the transcription of target genes.

EMT regulation during hypoxia is ensured predominantly by the HIF-1 and Snail/Slug factors. EMT induction under hypoxic conditions was shown in various tumor cell lines [77, 78]. Hypoxia decreases the expression of E-cadherin via a HIF-1α-mediated expression of *SNAI1*. In addition, HIF-1α induces LOX expression, which leads to the stabilization of Snail [79].

In response to hypoxia, the LOX protein level increases in tumor cells, and suppression of LOX expression/ activity prevents metastasis. A high LOX level is considered a factor of poor clinical prognosis associated with the metastasis of BC and head and neck cancers [80].

Yang and co-authors could demonstrate that HIF-1α regulates the activation of EMT, increasing the Snail level in gastric cancer stem cells. HIF-1α expression in these cells is significantly increased under conditions of hypoxia. As HIF-1α increases, the expression of Snail, vimentin, and N-cadherin is elevated, and the E-cadherin level decreases, which is an indication of EMT initiation. Under hypoxia, the possibility of migration and invasion of gastric cancer stem cells significantly increases [81].

We studied the relationship between β-catenin and Snail-dependent pathways in BC cells during hypoxia and found a Snail-dependent activation of β-catenin. Activated β-catenin regulates the expression of hypoxia- response genes and maintains a resistance of BC cells to reduced partial oxygen pressure. Coordinated activation of the Snail/β-catenin/HIF-1α protein system may be considered as an important factor in determining tumor resistance to hypoxia [82].

We showed that the HBL-100 BC cell line with Snail knockdown is more sensitive to hypoxia, demonstrating blockage of replication and a decrease in the percentage of mitotic cells. In addition, the culture density directly affects the sensitivity of BC cells to hypoxia [83].

Thus, responding to hypoxia, cells acquire a mesenchymal phenotype through EMT induced by HIF-1,2α and Snail/Slug. These phenotypic changes can be regulated by various epigenetic factors [76].

**Fig. 4:**
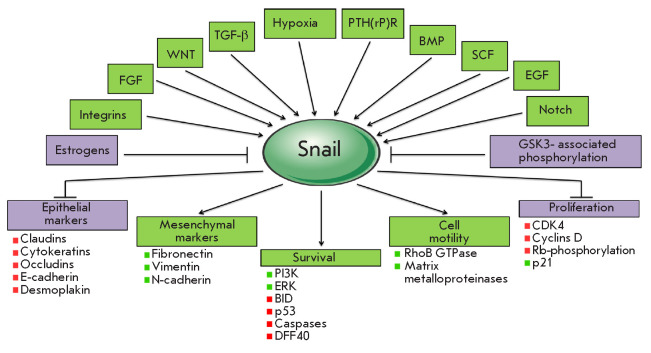
Regulation and main targets of the Snail transcription factor

*Figure 4* illustrates the regulation of numerous Snailmediated processes.

## EXPRESSION OF Snail-FAMILY PROTEINS IN TUMORS AS A POTENTIAL PROGNOSTIC MARKER

Snail and Slug are aberrantly expressed in many tumors, as well as in tumor-associated fibroblasts and macrophages that colonize damaged tissues [84, 85, 86]. Numerous studies have shown that these proteins play different roles in tumor progression.

**Table:** Expression of SNAI1 and SNAI2 and overall survival rate of cancer patients: analysis of data from the KM-plotter database

Tumor	Indicator			
	SNAI1*		SNAI2*	
	Total survival (median)	Statistical significance	Total survival (median)	Statistical significance
Bladder cancer	Low expression (exp) = 42.33 mos, high exp = 28.63 mos	P = 0.0264, q > 0.5	Low expression (exp) = 47.33 mos, high exp = 20.77 mos	P = 0.0008, q = 0.2
Squamous cervical cancer	Low exp = 68.4 mos, high exp = 27.9 mos	P = 0.027, q > 0.5	The difference is statistically insignificant	
Esophageal adenocarcinoma	Low exp = 46.83 mos, high exp = 20.33 mos	P = 0.0449, q > 0.5	The difference is statistically insignificant	
Squamous cell carcinoma of the head and neck	Low exp = 58.73 mos, high exp = 46.6 mos	P = 0.0398, q > 0.5	Low exp = 58.73 mos, high exp = 37.77 mos	P = 0.0174, q > 0.5
Clear cell renal cell carcinoma	Low exp = 73 mos, high exp = 37.03 mos	P = 0.0058, q > 0.5	Low exp = 52.8 mos, high exp = 37.03 mos	P = 0.0323, q > 0.5
Papillary renal cell carcinoma	Low exp = 89.47 mos, high exp = 43.53 mos	P = 8.2e–5, q = 0.02	Low exp = 86.97 mos, high exp = 43.8 mos	P = 0.0014, q > 0.2
Lung adenocarcinoma	Low exp = 50.93 mos, high exp = 40.3 mos	P = 0.0124, q > 0.5	Low exp = 54.4 mos, high exp = 35.77 mos	P = 0.0014, q > 0.5
Squamous cell lung cancer	Low exp = 72.33 mos, high exp = 35.83 mos	P = 0.0002, q = 0.05	The difference is statistically insignificant	
Ovarian cancer	Low exp = 49.97 mos, high exp = 38.97 mos	P = 0.0089, q > 0.5	Low exp = 46.13 mos, high exp = 38.7 mos	P = 0.0192, q > 0.5
Pancreatic ductal adenocarcinoma	The difference is statistically insignificant		Low exp = 37.67 mos, high exp = 18.93 mos	P = 0.0006, q = 0.2
Rectal adenocarcinoma	Low exp = 43.8 mos, high exp = 41.93 mos	P = 0.0384, q > 0.5	The difference is statistically insignificant	
Sarcoma	The difference is statistically insignificant		Low exp = 86.63 mos, high exp = 48.87 mos	P = 0.001, q = 0.2
Gastric adenocarcinoma	Low exp = 43.8 mos, high exp = 41.93 mos	P = 0.0384,>q > 0.5	Low exp = 46.9 mos, high exp = 20.23 mos	P = 0.0013, q = 0.2
Thyroid cancer	Low exp = not achieved, high exp = not achieved	P = 3.3–6, q = 0.01	The difference is statistically insignificant	
Uterine corpus cancer	Low exp = 114.1 mos, high exp = 51.6 mos	P = 0.0614, q = 0.01	Low exp = 36.87 mos, high exp = 78.4 mos	P = 0.0113, q ≥ 0.01

^*^The differences in the expression of SNAI1 and SNAI2 are statistically insignificant in esophageal squamous cell carcinoma, liver cancer, breast cancer, and uterine corpus endometrial cancer.

Expression of both *SNAI1 *and *SNAI2 *in tumor cells can characterize the degree of malignancy and serve as a prognostic marker of disease. Access to open sequence databases enables the use of various bioinformatics tools for a preliminary assessment of disease prognosis. A similar analysis is performed at the initial stage of the search and validation of new markers and clinically significant criteria. One of these databases, the KM-plotter, contains the gene expression profiles from the GEO, EGA, and TCGA databases [87]. The KM-plotter enables an assessment of the effect of gene expression on the overall survival rate of patients using the Kaplan–Meier method [88]. A total of 54,000 genes can be analyzed in 21 neoplasm types. The summarized data on the expression of *SNAI1 *and *SNAI2 *in tumors of each type are presented in *Table*. The analysis involved data on the expression of these genes in 19 neoplasm types; no statistically significant differences (in at least one of the indicators) in the overall survival rate were found for four of the genes. *SNAI1 *expression was shown to affect statistically significantly the median overall survival rate in 12 neoplasm types. The greatest difference in the median overall survival rate was found for squamous cervical cancer: the median survival rate was 2.4-fold higher in the group with a low *SNAI1 *expression than in the group with a high expression of this gene. These data are consistent with the results reported in a recent publication by Huilun Yang *et al*. [89], who proved the relationship between* SNAI1 *and *TWIST1 *and active metastasis of cervical cancer. In addition, these data were confirmed by a immunohistochemical analysis [90] of 154 cervical cancer samples. The smallest (significant) difference in the overall survival rate, depending on the *SNAI1* level, was found in gastric and rectal adenocarcinomas. It is noteworthy that *SNAI2 *expression does not affect overall survival indicators in rectal adenocarcinoma. The limited use of Snail as an individual (independent) prognostic marker of rectal cancer is indicated by the results of a study [91] that suggested combining EMT markers with stem cell markers to improve the predictive value of each individual indicator. Similar findings were obtained in a study of the relationship between *SNAI2 *expression and the overall survival rate in 10 tumor types. In most tumor types, a change in the *SNAI2 *expression has the same tendency as in the *SNAI1 *expression: high expression of the marker is considered a poor prognosis factor. An exception to this rule is uterine corpus cancer: high *SNAI2 *expression in this neoplasm is associated with longer overall survival. One of the explanations for this may be the low Slug activity in uterine corpus cancer cells. For example, a nuclear localization of Slug was established only in 3.7% of tumor samples; i.e., the clinical significance of this indicator is very limited [92]. Based on other data, 25% of uterine corpus cancer cases had high Slug expression; this indicator is associated with recurrence-free survival; therefore, it may be considered a poor prognosis factor [93]. The prognostic role of Slug (or its absence) in uterine corpus cancer remains to be clarified.

Despite the absence of statistically significant differences in the overall survival of BC patients in groups with different *SNAI1 *and *SNAI2 *levels (KM-plotter base), a number of studies have shown the clinical significance of EMT markers: in particular Snail, in this disease. In BC cells, there is a high expression of Notch (74%), Slug (36%), Snail (62%), and N-cadherin (77%), while the expression of E-cadherin is increased in just 20% of cases [68]. An analysis of 157 BC samples revealed a statistically significant correlation between the expression of Snail and Slug and their co-activator, the NF-κB factor [94]. According to Cao *et al*., high expression of Snail and a low level of E-cadherin correlate with the number of BC metastases in lymph nodes. In addition, a high level of Snail is largely associated with a low expression of E-cadherin, and an increased expression of Slug is associated with an increase in N-cadherin in BC patients [63].

The levels of Snail, Slug, and ZEB1 are higher in tumor cells with morphological signs of EMT (the ability to migrate and invade) than in cells without signs of EMT [95]. Knockdown of the *SNAI1 *and *SNAI2 *genes causes a return to an epithelial morphology and a significant decrease in the number of cells migrating in the Boyden chamber. Feng and co-authors showed that the levels of Snail, E-cadherin, Slug, and Twist – but not N-cadherin – were higher in malignant epithelial cells than in benign neoplasms [96].

A low level of E-cadherin expression and a high level of N-cadherin expression are characteristic of gastric cancer metastases and undifferentiated tumor cells, which correlates with a poor prognosis. A high expression of Snail in the primary tumor and a low expression in metastases correlate with further progression of metastasis and a negative prognosis [97].

A high expression of Snail, but not Slug, and low expression of E-cadherin are associated with poorer survival chances in bladder cancer [98]. In cervical cancer, an increase in Snail and a decrease in E-cadherin are negative prognostic factors. According to recent data, expression of Snail is a more significant predictor of this disease than the expression of other EMT regulators (Slug, ZEB1, and Twist) [99].

Overexpression of the epidermal growth factor receptor Her2/Neu stabilizes Snail, promoting drug resistance in gastric cancer [100] and BC [101]. In an inducible Her2/Neu- expressing BC model, Moody and co-authors found that the rate of tumor recurrence correlates with a high level of Snail [102]. Increased expression of Snail and Twist is associated with a poor prognosis for estrogen-positive BCs [103].

Therefore, the expression of EMT markers, in particular Snail family proteins, is associated with the degree of malignancy and, in general, with disease progression. It is reasonable to believe that the studied EMT markers can be prognostically significant in some cases [96]. But for implementation in clinical practice, it is necessary to choose analytical methods, validate them, and prove the economic feasibility of using new markers.

## Snail PROTEINS AND CHEMOTHERAPY RESISTANCE

EMT regulatory proteins can control not only the ability of tumor cells to invade and undergo metastasis, but also their resistance to genotoxic and targeted anticancer drugs. The mechanisms underlying this resistance are mediated by anti-apoptotic effects, decreased proliferation, and the emergence of multidrug resistance. The role played by Slug and Snail in the development of resistance to chemotherapy and radiotherapy has been shown in a number of studies [104].

For example, the Snail protein level is increased in cisplatin-resistant tumors and cell lines [105]. In addition, Snail induces gemcitabine resistance in pancreatic cancer [106] and BC [107] models and etoposide resistance in a small-cell lung cancer model [108].

Haslehurst and co-authors showed that expression of the *SNAI1*, *SNAI2*, *TWIST*, and *ZEB2 *genes is increased in the ovarian cancer A2780 cell line resistant to cisplatin. Cisplatin-resistant cells had a mesenchymal phenotype and lacked intercellular junctions, while sensitive cells retained epithelial morphology. Upon knockdown of the genes of key EMT regulators, Snail and Slug, cells returned to their initial epithelial phenotype in [42].

The stability of Snail under the action of cisplatin is due to deubiquitination of Snail by the USP1 protein that is induced upon DNA damage and stabilizes a number of repair and anti-apoptotic proteins [109]. Snail is similarly stabilized by TGF-β-activated USP27x deubiquitinase in a cisplatin resistance model [110]. The repair enzymes PARP-1 and PARP-3 are another mechanism of the relationship between DNA damage and Snail expression in response to chemotherapy. PARP-1 controls Snail expression at the transcriptional level in cells exposed to doxorubicin, and ABT-888, a PARP-1 inhibitor, is able to enhance the response of BC cells (MDA-MB-231 line) to doxorubicin. Inhibition of PARP-1 can increase tumor cell sensitivity *in vivo *by decreasing the expression of Snail [111]. Similarly, PARP-3 depletion inhibits the TGF-β- dependent EMT of BC cells, preventing the binding of Snail to E-cadherin and increasing their sensitivity to chemotherapy [112].

Snail-family transcription factors also mediate cell resistance to certain targeted drugs. Slug expression is increased in a lung cancer model resistant to gefitinib, an EGFR inhibitor, and in biopsies from patients treated with EGFR inhibitors. In this model, Slug repressed caspase-9 and pro-apoptotic protein Bim and suppression of Slug increased the sensitivity of cells to EGFR inhibitors [113]. Snail determines the resistance of triple-negative BC cells to rapamycin and everolimus, which are mTOR protein kinase inhibitors. In this model, trametinib, a histone deacetylase inhibitor, inhibited Snail-induced EMT in [102].

The role of Snail and Slug in the drug resistance of tumor cells is associated with a repression of the genes of the pro-apoptotic PUMA, ATM, PTEN, p53, BID, and caspase-6 proteins and de-repression of the genes of the proteins associated with the stemness phenotype [52, 55, 57]. Apart from its anti-apoptotic effect, Snail also increases the expression of ABC transporters, which are the most important mechanism of multidrug resistance [114].

Snail can regulate immune responses. For example, TGF-β induces Snail in macrophages migrating to the inflammation site or wound [81]. Tumors with a high level of Snail expression contain few infiltrating CD8^+^ cytotoxic T-lymphocytes and an increased amount of pro-tumor M2 macrophages [115]. Snail also induces immunosuppression and immunoresistance through cytokine TSP1 and TGF-β-activated regulatory T cells that reduce the expression of stimulating molecules in dendritic cells, which suppresses cytotoxic T lymphocytes [116].

Cancer stem cells (CSCs) are a small population of cells that are characterized by the expression of stemness markers and pluripotency. CSCs are believed to be a source of tumor heterogeneity. In particular, the tumor clonality maintained by the CSC population is a factor of chemo- and radioresistance. There is evidence that CSCs possess an increased metastatic potential, but the mechanisms of this process are not well understood [117, 118, 119]. The stemness regulator SOX2 induced by the vascular endothelial growth factor (VEGF-A) was shown to trigger EMT and metastasis. In BC lines and native tumor cells, VEGF-A activates SOX2 expression, which leads to *SNAI2 *induction through miR-452, EMT activation, and increased invasion and metastasis. Thus, VEGF-A stimulates SOX2- and Slugdependent invasion [120]. Therefore, overexpression of the EMT transcription factor Slug increases the migration activity of CSCs [96].

Activation of the SCF/c-Kit signaling pathway leads to an increase in the Slug level, which causes resistance of ovarian cancer cells to radiotherapy and promotes the survival of CSCs [57]. In addition, SCF/c-Kit/Slug mediates drug resistance in human mesothelioma cells. Knockdown of c-Kit/*KIT *or *SNAI2 *increases the sensitivity of mesothelioma cells to the chemotherapeutic agents doxorubicin, paclitaxel, and vincristine. Transfection of c-Kit/*KIT *into mesothelioma cells in the presence of SCF enhances Slug activity and increases resistance to these drugs. Mesothelioma cells with high Slug levels are resistant to drug therapy [121].

Therefore, Snail-family proteins can directly participate in the development of resistance to therapy and suppression of antitumor immunity. These properties of Snail, along with their involvement in EMT, indicate a need for pharmacological inhibition of these proteins.

## POTENTIAL OF PHARMACOLOGICAL INHIBITION OF Snail

**Fig. 5:**
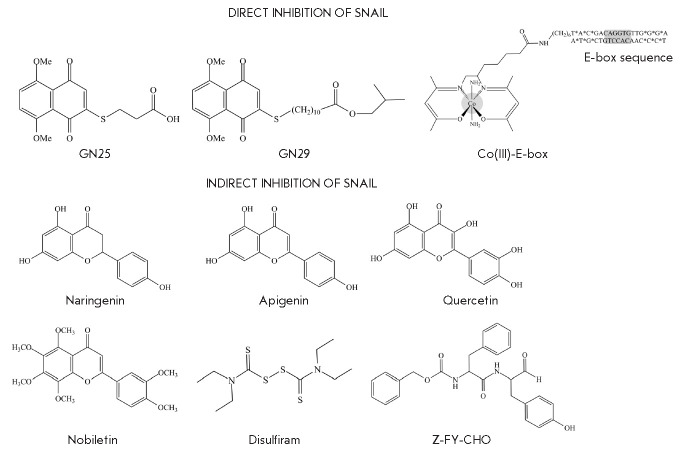
Pharmacological inhibitors of Snail functions

Signaling pathways involving Snail-family proteins are of interest in the search for new approaches in chemotherapy. Direct pharmacological inhibition is hindered by the complexity involved in targeting the protein’s functional domain. However, there have been successful attempts (*Fig. 5*).

Vistain *et al*. [122] proposed the E-box, a Snailbinding site, as a target. A Co(III) complex conjugated to a CAGGTG hexanucleotide was synthesized. After entering the cell, the Co(III)–E-box complex binds to Snail and prevents any interaction with DNA. The developed constructs significantly reduced the invasive potential of tumor cells. The authors hope this compound will be highly efficient as a therapeutic inhibitor of tumor progression and BC metastasis.

The search for a chemical inhibitor of Snail was carried out in [123]. The Snail–p53 complex was chosen as a target. A series of compounds were synthesized, and two leader compounds, GN25 and GN29, increasing the expression of p53 and uncoupling it from Snail were identified. Compounds GN25 and GN29 exhibited selectivity for K-Ras mutated cells and low toxicity for non-tumor cells. However, the effect of these compounds on tumor cells remains ambiguous and their mechanism of action is not well understood. So, it is too early to think about clinical trials of these compounds.

There are a number of compounds that affect the expression of Snail but are not its direct inhibitors. Disulfiram (DSF), which is used in the treatment of alcohol dependence, inhibits NF-κB. DSF inhibits TGF-β-induced EMT in BC cells, migration and invasion, and growth of tumor grafts. DSF inhibits the ERK/NF-κB/Snail signaling pathway, which leads to MET [124]. DSF is currently under Phase 2 clinical trials to treat patients with stage 4 BC in the Czech Republic. Z-FY-CHO, a selective inhibitor of cathepsin L (ECM component protease), was found to reduce the expression of Snail and trigger MET in prostate cancer cells with a mesenchymal phenotype [125].

MET can be initiated by phytoestrogens that modulate signaling through Snail and Twist1. The flavanone naringenin reduced the invasiveness of prostate cancer cells by blocking Snail and Twist1 [126]. Similar activity was reported for nobiletin, a flavonoid from citrus plants. This compound affects the signaling pathways of TGF-β, ZEB, Slug, and Snail and is capable of suppressing the invasion and migration of tumor cells [127]. The interest of researchers in potential EMT inhibitors of natural origin is justified by the relatively low toxicity of these compounds to non-tumor tissues, as well as by their anticarcinogenic properties [128, 129, 130]. Indeed, the flavonoid apigenin exerts an antiproliferative effect on BC cells with mesenchymal traits [131]. This phytoestrogen has been reported to suppress Snail expression, EMT, and cell metastasis [132, 133, 134]. Also, the flavonoid quercetin exhibits an antimetastatic effect [135]. Treatment of lung cancer cells with quercetin decreased their invasive and migratory activity. Quercetin affected the Akt-Snail signaling pathway that maintains the survival and metastatic ability of cells. Quercetin is currently under clinical trials as treatment for patients with prostate (phase 2), lung (not specified phase), and kidney (phase 2) cancers. To prevent EMT, it seems relevant to develop compounds that inactivate Snail family proteins and prevent the transactivation of their target genes.

The ability of these compounds to inhibit the functions and activity of Snail suggests that these compounds, after more detailed and thorough investigation of their mechanisms of action, may be included in clinical trials as agents to treat progressive and metastatic tumors.

At the moment, researchers are focused on modifying compounds, finding the best way to deliver them, and developing therapies in combination with other cytotoxic drugs [136].

## CONCLUSION

Snail family proteins are key EMT regulators that modulate many ontogenetic and neurobiological processes. A detailed investigation of EMT in tumor cells has revealed the important role played by this process in invasion and metastasis. Snail transcription factors are specific “switches” of the epithelial, more favorable, phenotype of cells to an aggressive prometastatic one. That is why molecular events mediated by these proteins are of interest as targets for therapy of, in particular, resistant metastatic tumors. The development of pharmacological approaches to Snail inhibition is in its infancy. However, chemical classes of synthetic and natural compounds affecting the transcriptional activity and expression of Snail and initiating MEP have already been characterized. Further investigation of EMT and its regulators appears promising for a personalized therapy of tumors.

## Abbreviations

ECM: extracellular matrix
MMP: matrix metalloproteinase
MET: mesenchymal-epithelial transition
TSCs: tumor stem cells
BC: breast cancer
EMT: epithelial-mesenchymal transition
